# Summarizing Complex Graphical Models of Multiple Chronic Conditions Using the Second Eigenvalue of Graph Laplacian: Algorithm Development and Validation

**DOI:** 10.2196/16372

**Published:** 2020-06-17

**Authors:** Syed Hasib Akhter Faruqui, Adel Alaeddini, Mike C Chang, Sara Shirinkam, Carlos Jaramillo, Peyman NajafiRad, Jing Wang, Mary Jo Pugh

**Affiliations:** 1 Department of Mechanical Engineering The University of Texas at San Antonio San Antonio, TX United States; 2 Department of Mathematics and Statistics University of the Incarnate Word San Antonio, TX United States; 3 South Texas Veterans Health Care System San Antonio, TX United States; 4 Department of Information Systems and Cyber Security The University of Texas at San Antonio San Antonio, TX United States; 5 School of Nursing UT Health San Antonio San Antonio, TX United States; 6 VA Salt Lake City Health Care System Salt Lake City, UT United States

**Keywords:** graphical models, graph summarization, graph Laplacian, disease network, multiple chronic conditions

## Abstract

**Background:**

It is important but challenging to understand the interactions of multiple chronic conditions (MCC) and how they develop over time in patients and populations. Clinical data on MCC can now be represented using graphical models to study their interaction and identify the path toward the development of MCC. However, the current graphical models representing MCC are often complex and difficult to analyze. Therefore, it is necessary to develop improved methods for generating these models.

**Objective:**

This study aimed to summarize the complex graphical models of MCC interactions to improve comprehension and aid analysis.

**Methods:**

We examined the emergence of 5 chronic medical conditions (ie, traumatic brain injury [TBI], posttraumatic stress disorder [PTSD], depression [Depr], substance abuse [SuAb], and back pain [BaPa]) over 5 years among 257,633 veteran patients. We developed 3 algorithms that utilize the second eigenvalue of the graph Laplacian to summarize the complex graphical models of MCC by removing less significant edges. The first algorithm learns a sparse probabilistic graphical model of MCC interactions directly from the data. The second algorithm summarizes an existing probabilistic graphical model of MCC interactions when a supporting data set is available. The third algorithm, which is a variation of the second algorithm, summarizes the existing graphical model of MCC interactions with no supporting data. Finally, we examined the coappearance of the 100 most common terms in the literature of MCC to validate the performance of the proposed model.

**Results:**

The proposed summarization algorithms demonstrate considerable performance in extracting major connections among MCC without reducing the predictive accuracy of the resulting graphical models. For the model learned directly from the data, the area under the curve (AUC) performance for predicting TBI, PTSD, BaPa, SuAb, and Depr, respectively, during the next 4 years is as follows—year 2: 79.91%, 84.04%, 78.83%, 82.50%, and 81.47%; year 3: 76.23%, 80.61%, 73.51%, 79.84%, and 77.13%; year 4: 72.38%, 78.22%, 72.96%, 77.92%, and 72.65%; and year 5: 69.51%, 76.15%, 73.04%, 76.72%, and 69.99%, respectively. This demonstrates an overall 12.07% increase in the cumulative sum of AUC in comparison with the classic multilevel temporal Bayesian network.

**Conclusions:**

Using graph summarization can improve the interpretability and the predictive power of the complex graphical models of MCC.

## Introduction

### Background

**Figure 1:**
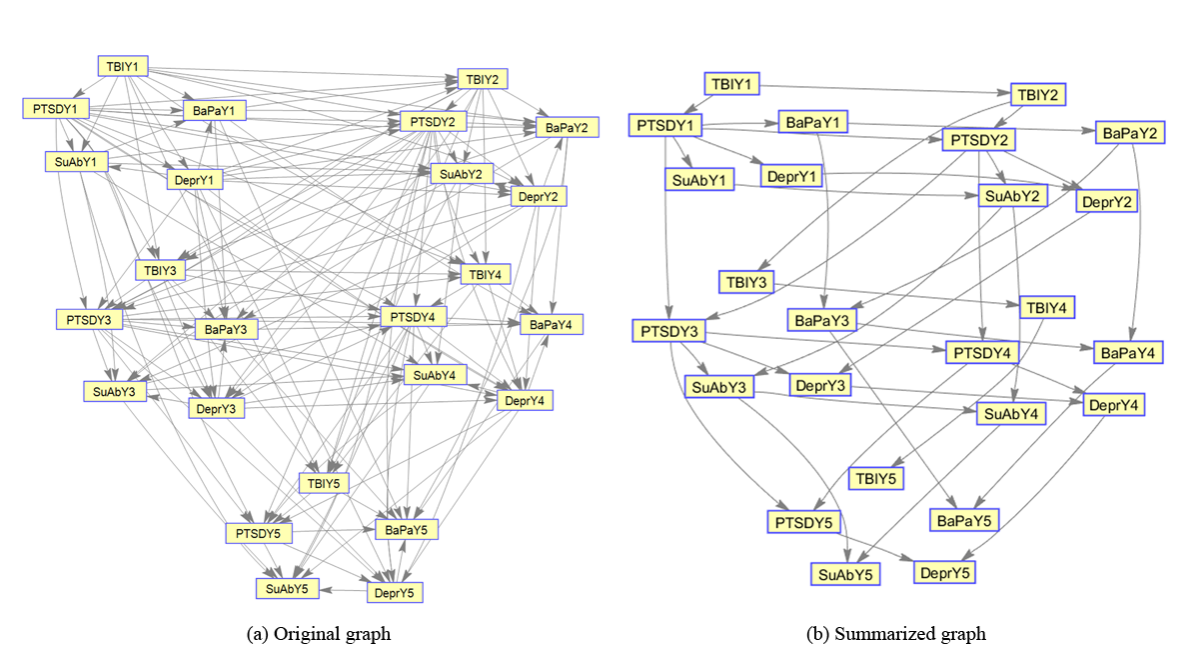
Learning sparse graphical models directly from emergence data on multiple chronic conditions using (a) the unsummarized graphical model (λ=0) and (b) the summarized graphical model using the EAGL structure learning algorithm (λ=1000) in which each node is a binary (0,1) variable representing the status (presence or absence) of a chronic condition in a particular year, that is, TBIY1 denotes the status of traumatic brain injury at year-1 (base year) and BaPaY5 denotes the status of back pain in year-5. BaPa: back pain; TBI: traumatic brain injury; PTSD: posttraumatic stress disorder; SuAb: substance abuse; MCC: multiple chronic conditions; EAGL: eigenvalue analysis of the graph Laplacian.

**Figure 2:**
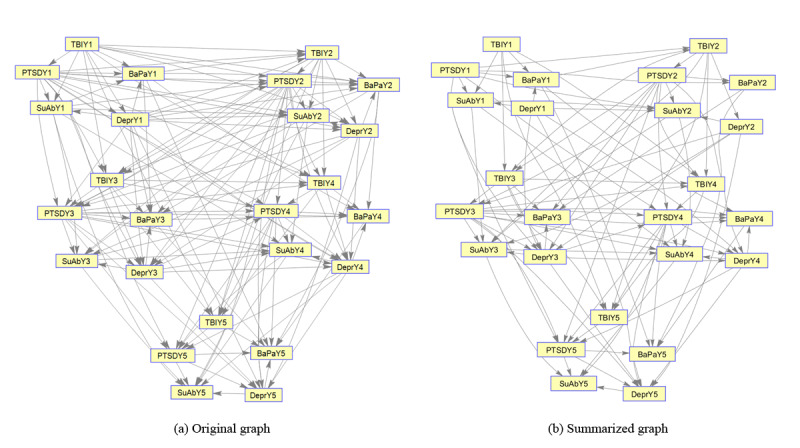
(a) Unsummarized probabilistic graphical model of the emergence of MCCs. (b) The summarized probabilistic graphical model using the EAGL summarization algorithm at a 20% summarization rate. Each node is a binary (0,1) variable representing the status (presence or absence) of a chronic condition in a particular year, that is, TBIY1 denotes the status of TBI at year-1 (base year), and BaPaY5 denotes the status of BaPa in year-5. BaPa, back pain. In the figure, BaPa: back pain; TBI: traumatic brain injury, PTSD: posttraumatic stress disorder and SuAb: substance abuse, MCC: multiple chronic condition, EAGL: eigen analysis of graph Laplacian.

Clinical data on multiple chronic conditions (MCC) are often complex [1-4] and large [5-8]. These challenging data sets can be effectively represented in terms of graphical models [4,9]. A graphical model expresses the conditional dependencies among variables (MCC) using graph structures, where the dependencies are represented by directed or undirected edges and the variables are represented by nodes [10,11]. Analyzing these graph structures enables us to get an insight into the interactions among different chronic conditions as well as the path toward developing MCC [12]. Graphical models can also be used for the (quantitative) prediction of the occurrence versus nonoccurrence of new chronic conditions over time, based on the existing conditions, sociodemographic factors, and so on [4,13-15]. With the advancement of medical technology, the amount of data collected from different electronic medical records systems is increasing. Thus, such disease interaction graphs are becoming larger and more complex. For example, a graphical model to characterize the interaction among 30 MCC over time requires more than 1 billion edges to investigate, or a temporal graphical model to represent the relationship among 5 MCC over 5 years (time stages) requires over 400 edges to explore. There are also numerous examples of complex networks in gene expression and molecular analysis [8,16,17]. However, a large graph may have less significant edges or noisy connections, which will affect the accuracy of analysis and slow down the learning and prediction process in big data settings. Such an unsummarized graph is shown in Figure 1 (and Figure 2). Meanwhile, medical practitioners often need more concise representation to interpret the results, such as understanding the major evolution paths of MCC for planning proper intervention [9,18].

Thus, instead of using a fully/densely connected network for analysis, choosing a network with fewer but more informative connections can improve the training and querying process. However, the main questions are as follows: (1) What are the least/most informative parts of the graphical models? (2) How can such information be leveraged to summarize graphical models without losing considerable predictive/inference accuracy? and (3) How can an algorithm of this type be applied to learn a compact graph directly from the data? Effective summarization algorithms are the ones that preserve the most important structures of the original graphical model, focus on major patterns/aspects of the data, and maintain the original graph distribution (the conditional probability distribution of the original graph). They should also be capable of querying or identifying substructures/patterns in a specific set of nodes/triads (local queries) of the graph structures as well as the complete graph (global queries) to study the global influence of conditioned states.

Graph summarization is also affected by factors such as data volume and complexity (structure, heterogeneity, and abstraction), dynamic/static nature of the graph, efficiency of the inference procedure, and computational complexity of the summarization approach [19]. Existing graph summarization approaches can be divided into 5 major categories:

1. Clustering-based approaches, which aggregate nodes into super-nodes and connect them using super-edges, including spectral clustering [20-22], coclustering [23], cross association [24], shingle ordering [25,26], GraSS [27], and COARSENET [28].
2. Community-based approaches, which aggregate all the nodes that belong to the same community and superimpose edge weights by summing up the weights of the original edges [29-32].
3. Simplification-based approaches, which remove less important nodes/edges, including OntoVis [33], EgoCentric [34], and MDL-based approaches [35-38].
4. Pattern set mining approaches, which create subgraphs based on the extracted patterns, including VNM [39], SUBDUE [40], VoG [35], Oddball [41], and Pegasus [42].
5. Node/edge immunization/deletion approaches, which select the best flow of the information from the source to the destination node, including MIOBI [43] and NetMelt [44].

### Objective

**Figure 3:**
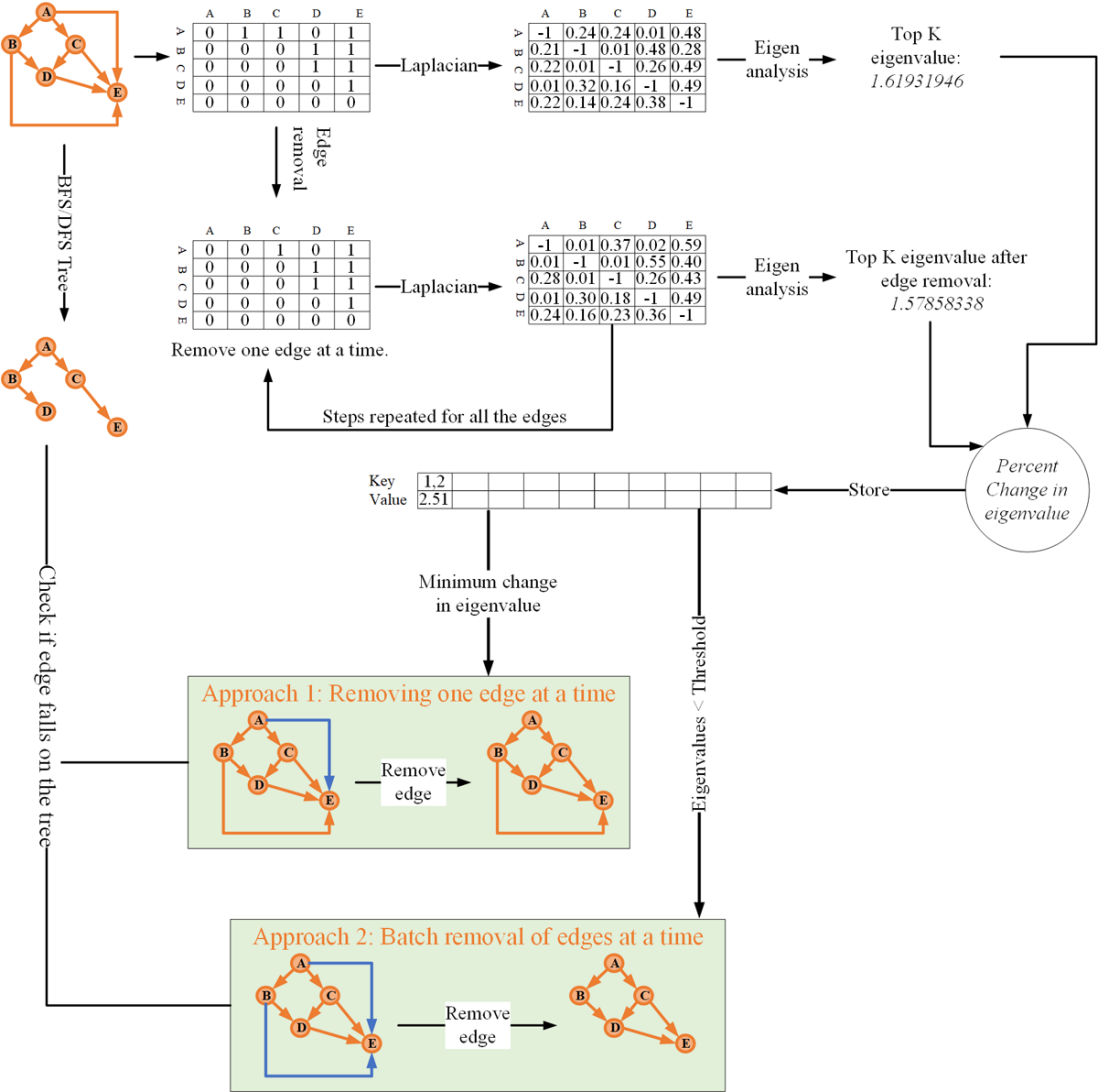
Visual representation of the proposed EAGL Algorithm for summarizing a directed probabilistic graphical model based on an available dataset.

In this work, we propose a graph summarization approach that utilizes the second eigenvalue analysis of the graph Laplacian (EAGL) to identify and prune less informative edges of the complex graphical models of MCC interactions. The intuition behind the proposed EAGL criterion is that the eigenvalue of the graph Laplacian of a graphical model is an effective measure of the connectivity and information flow [45,46]. The eigenvalue of the graph Laplacian also captures graph robustness, clustering coefficient, node importance, and several other properties [47,48]. The proposed simplification method can be utilized to (1) learn a sparse graphical model of MCC interactions directly from the data by adding a regularization term to an existing score-based structure learning algorithm to achieve a desired level of sparsity or (2) summarize a given graph of MCC interactions by removing less significant edges (with or without supporting data set) to speed up the inference process without sacrificing the predictive accuracy considerably (Figure 3). We applied the proposed approach to study conditional relationships (dependencies) among 5 multiple chronic medical conditions, including posttraumatic stress disorder (PTSD), traumatic brain injury (TBI), depression (Depr), back pain (BaPa), and substance abuse (SuAb), as well as most commonly related (coappeared) terms in the literature of MCC.

## Methods

### Probabilistic Graphical Models

A probabilistic graphical model is specified as a tuple, *B* = (*G,P*), where *G* denotes a graph that may be directed acyclic (in Bayesian networks, BN) or undirected (in a Markov random field), and *P*(*X_1_,X_2_,… …,X_k_*) denotes the joint probability distribution defined by conditional probabilities of the form *P*(*X = x_k_*|*Pa*(*X = x_k–1_*)), where *X* (upper case) denotes the conditional variables, *x* (lower case) denotes the associated values of the conditional variables, and *Pa*(*X = x_k–1_*) denotes the parents of a *X* [9-11,49-52]. *G* (*V,E*) consists of vertices (*V*), that is, MCC conditions, and arcs/edges (*E*), that is, MCC interactions/connections, corresponding to the random variables of consideration. The network represents the joint distribution over the random variables/nodes, which can be factored according to the dependencies represented in the graph, resulting in the decomposition property of the BN:

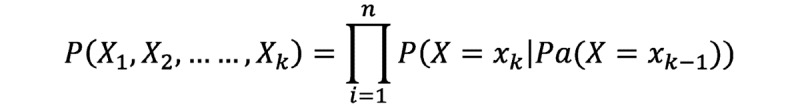



The decomposition property makes the Bayesian inference process simple. This model is also known as the recursive model. Here, we use binary variables (nodes) representing having or not having a chronic condition (TBI, PTSD, BaPa, Depr, and SuAb) for the probabilistic graphical models.

### Graph Laplacian

The graph Laplacian is a matrix representation of a graph, which can be used to study various properties of a graph. The first and second smallest eigenvalue of the graph Laplacian can be used to extract useful information such as graph communities (first smallest eigenvalue) and sparsest cut in a graph (second smallest eigenvalue) [45,53]. For an undirected graph, *G* (*V,E*), the graph Laplacian *L*(*G*) is defined as *L* = *D*–*A*, where *A* is the adjacency matrix, *D* is the degree matrix, and the elements of *L* are defined as follows [45,54]:

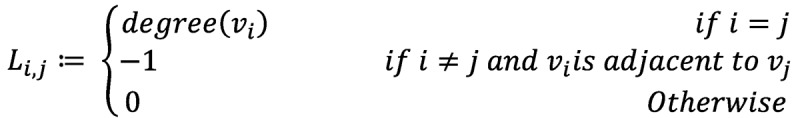



For a directed graph, we can consider both in- and out-degree to form the degree matrix [55,56]. In this work, we used the algorithm proposed by Fan et al [56] for deriving the graph Laplacian of directed graphical models, which is one of the most prominent methods in the literature and is straightforward to implement.

### Summarizing While Learning the Structure of the Probabilistic Graphical Models Directly From Data

**Figure 4:**
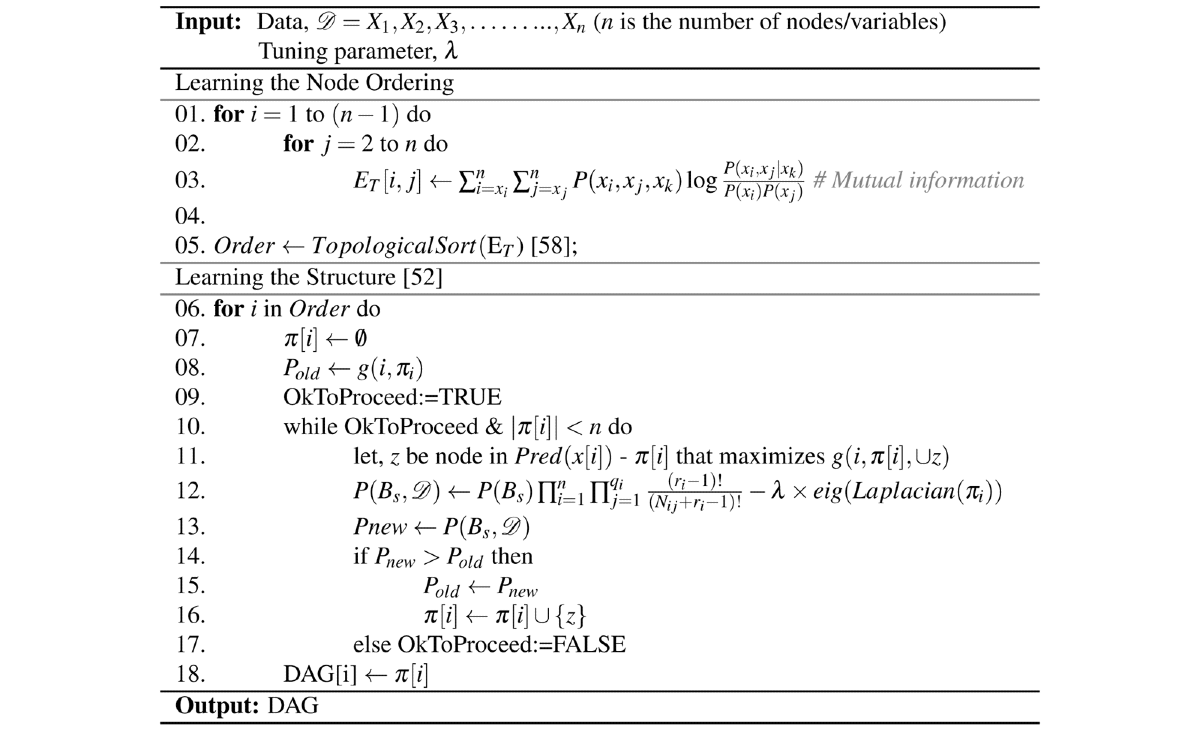
Algorithm for summarizing while learning the structure of the probabilistic graphical models directly from data.

Figure 4 presents the major steps of the proposed EAGL algorithm for learning the sparse probabilistic graphical model structure directly from the data. The algorithm utilizes an iterative score-based method (K2, min-max hill-climbing, etc) to learn the edges (relationship) between nodes [49,57] while incorporating an active learning regularization term based on the second eigenvalue of the Laplacian of the adjacency matrix (graph Laplacian) of the graph from its previous iteration to penalize for the inclusion of less informative edges. The size of the regularization term is controlled by changing the tuning parameter λ to achieve the desired level of sparsity. In this paper, we considered the *maximum weight spanning tree (MWST) + K2* algorithm as the base learning algorithm along with the second eigenvalue of the graph Laplacian to learn a sparse structure for the probabilistic graphical model from the data. For a given data set, the MWST algorithm [50] is used to learn the initial node ordering [58]*.* Utilizing the ordered nodes, a greedy search method such as K2 algorithm incrementally learns the directed acyclic graph (DAG) structure from the data [52]. The regularization term is added to the K2 score function to learn the sparse representation of the DAG structure. The analysis of the computation complexity of the EAGL algorithm is provided in the *Computational Complexity* subsection.

### Summarizing an Existing Probabilistic Graphical Model With Supporting Data

**Figure 5:**
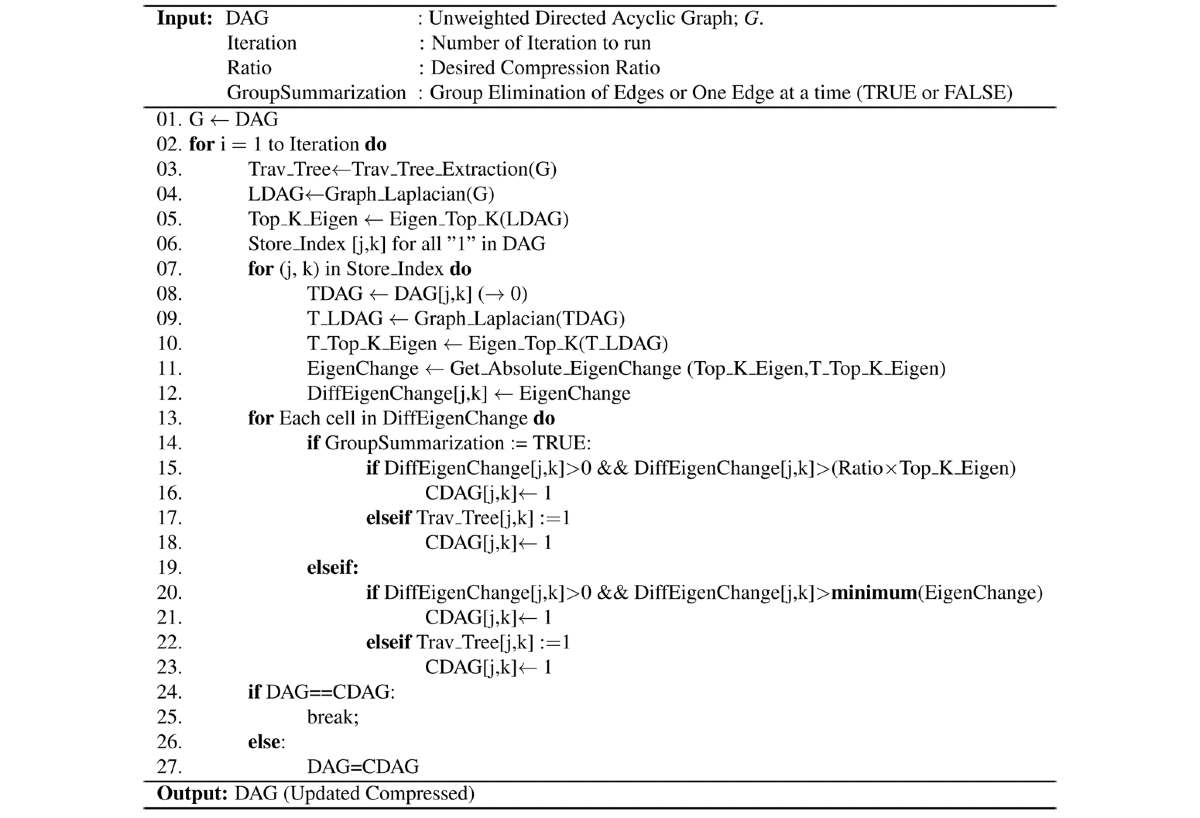
Algorithm for summarizing an existing probabilistic graphical model with supporting data.

Figure 5 presents the major steps of the proposed EAGL algorithm for summarizing probabilistic graphical models when a supporting data set is available. The algorithm starts with a given probabilistic graphical model and drops edges one at a time while monitoring the changes in the second eigenvalue of the graph Laplacian. Then, it prunes the edge/s with minimum changes (removal) in the second eigenvalue of the graph Laplacian. There are 2 possible strategies for pruning the edges: (1) single edge removal—where at each stage it prunes the edge with the minimum change in the second eigenvalue—and (2) multiple edge removal—where at each stage it prunes all the edges whose change in second eigenvalue is less than a preset value (eg, 0.05). The algorithm then stops when further pruning the remaining edges change will result in a significant change in the second eigenvalue (ie, >0.05). Once all the noninformative edges have been pruned, the conditional dependencies are updated based on the supporting data. The analysis of the computation complexity of the algorithm is provided in the *Computational Complexity* subsection.

### Summarizing an Existing Graphical Model Without Supporting Data

Excluding the step/s to update the remaining conditional dependencies in Figure 5 (after dropping each edge) will result in the summarization algorithm with no supporting data (see the subsection *Summarizing a Graphical Model of Multiple Chronic Conditions Terms With No Supporting Data* for results).

### Structural Constraints

To avoid creating isolated nodes or islands (cluster of isolated nodes) that affect the accuracy of inference and prediction (especially in temporal graphical models), we use graph traversal methods, specifically depth-first search (DFS) [59] to preserve a path between the root and leaf nodes (for information passing between nodes). The path attained from the graph traversal is considered as a constraint in the EAGL algorithm.

### Dynamic Graph

Considering the consecutive time instances of the dynamic graph, that is, t and t + 1, as a static graph, and applying appropriate structural constraints as discussed above, that is, DFS, the EAGL algorithm can be used to summarize dynamic graphical models as well.

## Results

### Study Population

The relationship among the emergence of MCC can be expressed effectively using probabilistic graphical models, where nodes represent the emergence of chronic conditions, that is, BaPa, Depr, and so on, and edges show the statistical relationship (conditional dependency) between them (BaPa and Depr). Here, we are interested in sparse learning of the structure and parameters of the probabilistic graphical model using the EAGL algorithm based on an available data set of the emergence of MCC. Our deidentified data were collected from a large national cohort of US military veteran patients (N=608,503), who were deployed in support of the wars in Afghanistan and Iraq and began receiving care in the Veterans Health Administration (VA) between 2002 and 2011. For the purpose of this analysis, we have only considered patients who received care each year for the first 5 years after entering VA care (N=257,633). Dropout may result from not requiring care, dropping out of VA care, or death. This study received institutional review board approval from the University of Texas Health Science Center at San Antonio and the Bedford VA Hospital, with a waiver of informed consent. A summary of the study population is shown in Table 1.

**Table 1:** Demographics of the patients included in the study.

Demographics		Serial number					
		1	2	3	4	5	6
Race		White	Black	Hispanic	Asian	Native	Unknown
**Gender, n (%)**							
	Male	148,355 (57.58)	35,758 (13.88)	25,373 (9.85)	5639 (2.19)	3081 (1.20)	2135 (0.83)
	Female	19,183 (7.45)	11,828 (4.59)	4232 (1.64)	981 (0.38)	707 (0.27)	361 (0.14)
**Marital status, n (%)**							
	Married	74,487 (28.91)	23,308 (9.05)	14,523 (5.64)	3067 (1.19)	1747 (0.68)	1346 (0.52)
	Unmarried	93,051 (36.12)	24,278 (9.42)	15,082 (5.85)	3553 (1.38)	2041 (0.79)	1150 (0.45)
**Age group (years), n (%)**							
	18-30	96,799 (37.57)	20,047 (7.78)	17,016 (6.60)	3235 (1.26)	2115 (0.82)	1062 (0.41)
	31-40	36,003 (13.97)	12,468 (4.84)	6606 (2.56)	1361 (0.53)	925 (0.36)	625 (0.24)
	41-50	26,167 (10.16)	12,710 (4.93)	4758 (1.85)	1564 (0.61)	564 (0.22)	673 (0.26)
	≥51	8569 (3.33)	2361 (0.92)	1225 (0.48)	460 (0.18)	184 (0.07)	136 (0.05)
**Education, n (%)**							
	Unknown	2334 (0.91)	658 (0.26)	386 (0.15)	131 (0.05)	60 (0.02)	51 (0.02)
	Less than high school	2037 (0.79)	504 (0.20)	360 (0.14)	60 (0.02)	60 (0.02)	22 (0.01)
	High school graduate	129,921 (50.43)	37,506 (14.56)	23,592 (9.16)	4732 (1.84)	3004 (1.17)	1808 (0.70)
	Some college	16,743 (6.50)	4819 (1.87)	2933 (1.14)	598 (0.23)	376 (0.15)	287 (0.11)
	College graduate	12,024 (4.67)	3160 (1.23)	1893 (0.73)	879 (0.34)	217 (0.08)	223 (0.09)
	Post college education	4479 (1.74)	939 (0.36)	441 (0.17)	220 (0.09)	71 (0.03)	105 (0.04)

### Learning Sparse Probabilistic Graphical Models Directly From Data

The EAGL algorithm begins with a DAG structure provided by a score-based algorithm [9,49], that is, MWST + K2. It then calculates the second eigenvalue of the graph Laplacian for the obtained DAG. Next, it multiplies the second eigenvalue with a tuning parameter. It adds it as a penalty term to the main scoring function to determine which edges to remove for the next iteration. The last 2 steps are repeated until a stopping criterion is met.

Figure 1 illustrates 2 graphical models, which have been estimated with different choices of the tuning parameter (λ) to control the sparsity in the EAGL algorithm: (1) the unsummarized graphical model without a penalty (λ=0) and (2) a summarized graphical model with a large tuning parameter (λ=1000). The tuning parameter was set at λ=0 (Figure 4), which results in an unsummarized graphical model [9] that provides a year 2 predictive accuracy of TBI=75.69%, PTSD=78.97%, BaPa=63.16%, SuAb=72.93%, and Depr=68.24%, compared with 72.34% reduction in the number of edges, and year 2 predictive accuracy of TBI=79.91%, PTSD=84.04%, BaPa=78.83%, SuAb=82.50%, and Depr=81.47% for the summarized graphical model (λ=1000; Figure 1, summarized graph; Table 2).

To evaluate the model, the area under the curve (AUC) of the receiver operating characteristic (ROC) curve [60] was considered. ROC curves are tools used to illustrate the diagnostic ability of a binary classifier at different threshold values. The curves are created by plotting the true positive rate (probability of detection) against the false positive rate (false detection ratio) at the threshold settings. This plot can be summarized into a single metric by calculating the area under the ROC curve. The AUC identifies how much a model is capable of distinguishing between different classes. AUC values range between 0 and 1, with higher values representing better classification accuracy. Table 2 illustrates the predictive accuracy of the learned graphical model under different choices of tuning parameters λ=0,10^–2^,10^–1^,...,10^5^ (λ=0 represents the classical/unsummarized graphical model) using the AUC metrics based on 10-fold cross-validation. It also shows the predictive performance of the learned graphical model using the popular Akaike information criterion (AIC). The superior predictive accuracy of the sparse graphical model by the EAGL algorithm can be attributed to the removal of spurious (less significant edges) edges in the graph, which improves the information propagation through high-confidence paths on the graph. Table 2 also compares the performance of the EAGL with another popular approach, AIC, which achieves 66.67% edge removal and year 2 predictive accuracy of TBI=59.49%, PTSD=63.45%, BaPa=78.51%, SuAb=61.32%, and Depr=59.05%.

**Table 2:** The area under the curve performance of the sparse probabilistic graphical model learned by the eigenvalue analysis of the graph Laplacian algorithm directly from the data with different choices of tuning parameters (λ=0,10^–2^,10^–1^,...,10^5^) for predicting future comorbidities (year 2 to year 5), given the comorbidity information of the past year (year 1), along with the area under the curve performance of a comparing algorithm, namely, Akaike information criterion (AIC) as well as the associated summarization ratios.

Prediction year		Lambda									
		0.00	0.01	0.10	1.00	10.00	100.00	1000.00	10000.00	100000.00	AIC
**Year 2 (%)**											
	TBI^a^	75.69	75.69	75.69	75.88	76.69	79.57	79.91	79.88	79.88	59.49
	PTSD^b^	78.97	78.97	79.08	79.53	81.31	83.11	84.04	83.70	83.70	63.45
	BaPa^c^	63.16	63.16	63.16	63.63	48.57	78.29	78.83	78.82	78.82	78.51
	SuAb^d^	72.93	72.93	73.04	73.33	75.22	74.58	82.50	85.00	85.00	61.32
	Depr^e^	68.24	68.24	68.27	68.45	71.02	74.26	81.47	81.61	81.61	59.05
**Year 3 (%)**											
	TBI	72.22	72.22	72.19	72.63	74.82	76.28	76.23	76.11	76.11	62.28
	PTSD	76.01	76.01	76.02	76.84	78.71	80.11	80.61	80.35	80.35	61.95
	BaPa	60.92	60.92	60.98	61.82	70.07	73.15	73.51	73.84	73.84	73.27
	SuAb	70.80	70.80	70.82	71.02	73.62	68.51	79.84	81.13	81.13	61.83
	Depr	65.16	65.16	65.18	65.93	69.01	70.48	77.13	77.10	77.10	56.09
**Year 4 (%)**											
	TBI	70.86	70.86	70.81	71.11	72.96	73.20	72.38	72.39	72.39	60.71
	PTSD	73.21	73.21	73.35	74.11	75.97	78.00	78.22	77.84	77.84	61.97
	BaPa	61.81	61.81	61.82	62.50	69.96	72.98	72.96	72.61	72.61	72.84
	SuAb	68.97	68.97	68.82	68.34	70.88	74.96	77.92	79.64	79.64	60.72
	Depr	64.73	64.73	64.79	65.09	67.29	68.00	72.65	73.54	73.54	56.23
**Year 5 (%)**											
	TBI	70.88	70.88	70.92	71.78	72.50	73.47	69.51	69.38	69.38	59.70
	PTSD	72.72	72.72	72.88	73.43	75.21	76.50	76.15	74.86	74.86	60.59
	BaPa	53.46	53.46	53.41	54.09	69.63	72.64	73.04	68.52	68.52	72.30
	SuAb	63.73	63.73	63.74	61.34	63.46	73.65	76.72	77.26	77.26	61.46
	Depr	64.01	64.01	64.11	64.87	66.58	67.89	69.99	71.37	71.37	56.07
**Edge details**											
	Edges, n	141	140	139	128	107	73	39	24	24	47
	Edge removal (%)	0.00	0.71	1.42	9.22	24.11	48.23	72.34	82.98	82.98	66.67

^a^TBI: traumatic brain injury.

^b^PTSD: posttraumatic stress disorder.

^c^BaPa: back pain.

^d^SuAb: substance abuse.

^e^Depr: depression.

**Figure 6:**
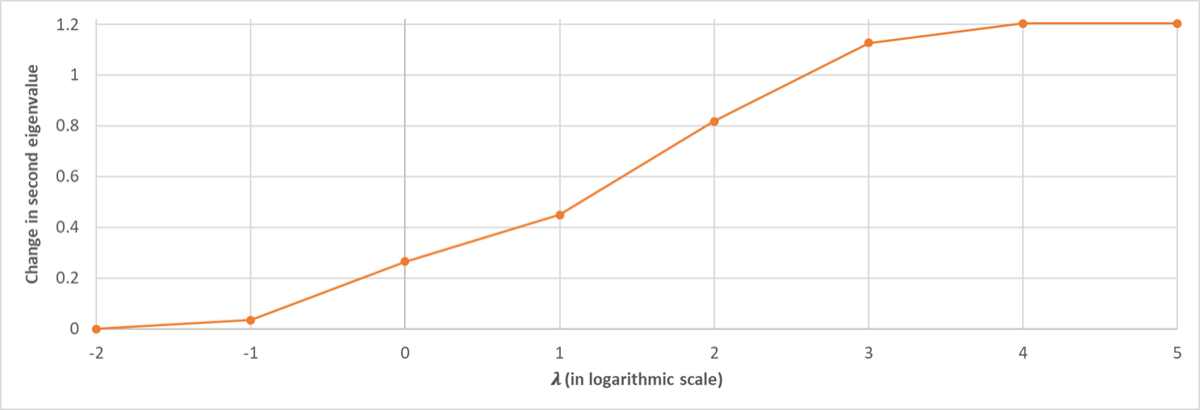
The relationship between the change in the tuning parameter (λ) and the second eigenvalue.

**Figure 7:**
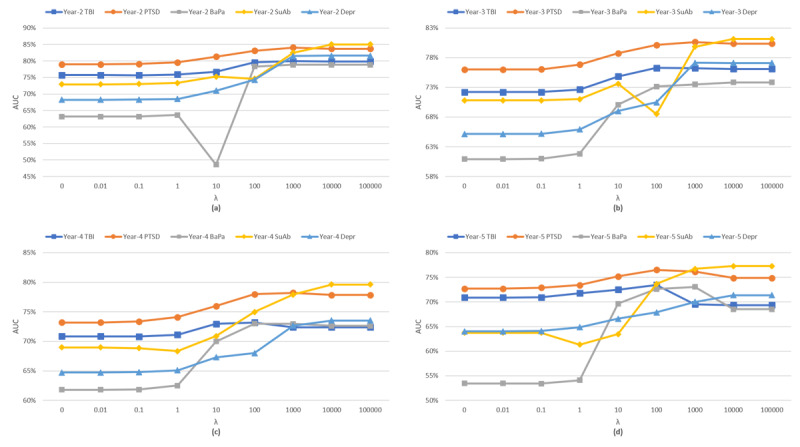
The relationship between the change in the tuning parameters (λ) and the area under the curve: (a) year-2; (b) year-3; (c) year-4; (d) year-5 of the study. BaPa: back pain; TBI: traumatic brain injury, PTSD: posttraumatic stress disorder and SuAb: substance abuse, MCC: multiple chronic conditions, EAGL: eigen analysis of graph Laplacian.

Figure 6 studies the relationship between the changes in the tuning parameters and the second eigenvalue of the graph Laplacian, which shows no change (in the second eigenvalue) over very small/large choices of the tuning parameters and logarithmic growth over other (midrange) choices of the tuning parameter. From Figure 7, we observed a similar pattern between changes in the tuning parameters and model sparsity and predictive accuracy, where very small (<0.01) or very large (>10^4^) changes in the tuning parameter did not improve the edge removal rate and/or predictive accuracy. Meanwhile, other choices of tuning parameters generally improve both sparsity and predictive accuracy. Therefore, the change in the second eigenvalue of the graph Laplacian can be used as a stopping criterion for EAGL algorithm; specifically, when increasing the tuning parameter does not change the second eigenvalue of the graph Laplacian, the algorithm shall stop (the analysis of first eigenvalue is provided in Multimedia Appendix 1).

### Summarizing an Existing Probabilistic Graphical Model With Supporting Data

In many real-life situations, we are given a graphical model that could potentially be simplified. The EAGL algorithm, which is based on the second eigenvalue of the graph Laplacian, can be used to identify and prune insignificant edges of the graph to achieve the desired level of summarization. The EAGL algorithm begins by calculating the second eigenvalue of the graph Laplacian of the given graphical model. It then extracts the DFS tree to determine the edges to avoid isolated nodes. Next, from the set of edges that is not lying on the DFS tree, the algorithm (temporarily) removes edges one at a time and calculates the percentage of the change in the second eigenvalue of the remaining graph Laplacian. Subsequently, it (permanently) removes the edge, resulting in a minimum change in the second eigenvalue of the graph Laplacian. The last 2 steps are repeated until a stopping criterion is met. Once the summarized network structure is attained, the weight S of the edges (conditional probabilities) are estimated using a standard parameter estimation algorithm [10,50]. Figure 3 provides a visual representation of the proposed algorithm. An example of this step-by-step process is provided in Multimedia Appendix 2.

Here, we are interested in summarizing an existing probabilistic graphical model of MCC relationships attained using a score-based method [9] based on the MCC data set discussed above (Figure 2, original graph). The summarized graph in Figure 2 illustrates the structure of the summarized graphical model based on removing less significant edges/paths of the original graphical model using the EAGL algorithm at a 20% summarization rate (removing 20% of existing edges).

Table 3 presents the AUC performance of the summarized graphical models at different summarization ratios of 0%, 1%, 5%, 10%, and 20% (0% represents the classical/unsummarized graphical model) for predicting future comorbidities (year 2 to year 5), given the year 1 comorbidity using 10-fold cross-validation. It also shows the predictive performance of the learned graphical model using the MIOBI [43] algorithm and the CHEETAH [61] algorithm at different summarization ratios. As shown in the table, the proposed EAGL algorithm generally provides the most competitive predictive accuracy among the comparing methods across different summarization ratios. This is while the EAGL algorithm also prevents the creations of island nodes, which helps with the interpretation of the results.

Although increasing the summarization ratio generally results in a sparser graphical model, for mild summarization ratios (<10%), using EAGL can also improve the predictive performance of the graphical model by preserving more informative edges/paths as it should. However, a large choice of summarization ratios (>10%) can decrease the predictive performance, depending on the topological location of the node (chronic conditions) and the associated edges that have been pruned (Table 3).

**Table 3:** The area under the curve performance of the original and summarized probabilistic graphical models at different summarization ratios (1%, 5%, 10%, and 20%) for predicting future comorbidities (year 2 to year 5), given the comorbidity information of the past year (year 1).

Prediction year		EAGL^a^						MIOBI [33]						CHEETAH [62]				
		Original	1.00	5.00	10.00	20.00	Original		1.00	5.00	10.00	20.00	Original		1.00	5.00	10.00	20.00
**Year 2 (%)**																		
	TBI^b^	75.69	75.63	75.53	75.09	63.34		75.69	75.78	75.63	63.99	56.08		75.69	75.70	65.25	63.95	61.80
	PTSD^c^	78.97	78.94	80.20	80.87	81.51		78.97	79.32	80.57	70.86	71.04		78.97	79.12	79.54	80.19	71.15
	BaPa^d^	63.16	63.18	61.05	61.37	65.53		63.16	62.99	63.14	62.44	63.25		63.16	63.20	63.43	64.16	64.80
	SuAb^e^	72.93	72.95	75.74	70.59	68.26		72.93	72.93	72.88	73.54	69.99		72.93	73.10	73.78	73.96	74.29
	Depr^f^	68.24	68.23	70.48	62.88	59.27		68.24	68.24	68.03	66.20	55.50		68.24	68.36	68.51	68.74	70.22
**Year 3 (%)**																		
	TBI	72.22	72.25	72.15	72.13	71.78		72.22	72.24	72.36	70.20	59.82		72.22	72.22	64.62	63.54	61.33
	PTSD	76.01	75.97	77.37	77.81	78.57		76.01	76.40	77.89	75.34	76.60		76.01	76.15	76.90	76.78	69.36
	BaPa	60.92	60.98	59.32	59.49	61.88		60.92	60.96	61.12	61.34	58.44		60.92	61.04	61.29	61.49	61.80
	SuAb	70.80	70.81	72.49	70.54	67.58		70.80	70.73	70.72	70.70	71.08		70.80	70.83	71.53	71.73	71.97
	Depr	65.16	65.20	66.95	68.29	58.28		65.16	65.13	65.16	64.45	64.24		65.16	65.37	65.84	65.99	66.85
**Year 4 (%)**																		
	TBI	70.86	70.78	71.10	70.76	70.48		70.86	70.82	70.82	69.41	68.20		70.86	70.27	64.85	64.08	61.94
	PTSD	73.21	73.18	74.29	74.95	76.02		73.21	73.52	74.99	72.92	74.51		73.21	73.34	73.88	74.11	67.64
	BaPa	61.81	61.81	60.24	60.38	63.01		61.81	61.76	62.01	62.62	59.29		61.81	61.93	62.14	62.43	63.03
	SuAb	68.97	69.00	70.28	69.13	68.24		68.97	68.97	68.73	69.28	70.99		68.97	69.14	69.53	69.78	70.24
	Depr	64.73	64.71	65.48	65.53	61.55		64.73	64.73	64.63	63.97	64.03		64.73	64.88	65.09	65.25	65.68
**Year 5 (%)**																		
	TBI	70.88	70.91	71.55	71.63	70.74		70.88	70.82	70.91	70.53	69.14		70.88	70.52	66.02	65.25	63.35
	PTSD	72.72	72.70	73.55	73.75	74.34		72.71	72.95	73.91	72.24	73.61		72.71	72.85	73.19	73.39	67.30
	BaPa	53.46	53.49	51.20	50.58	50.67		53.46	53.21	53.11	53.03	49.26		53.46	53.51	53.50	53.73	53.01
	SuAb	63.73	63.76	64.35	62.59	59.70		63.73	63.61	63.46	63.07	61.47		63.73	63.90	63.99	63.87	64.12
	Depr	64.01	63.99	64.78	64.50	61.23		64.01	63.96	63.74	63.38	63.32		64.01	64.19	64.50	64.70	65.19

^a^EAGL: Eigenvalue analysis of the graph Laplacian.

^b^TBI: traumatic brain injury.

^c^PTSD: posttraumatic stress disorder.

^d^BaPa: back pain.

^e^SuAb: substance abuse.

^f^Depr: depression.

**Figure 8:**
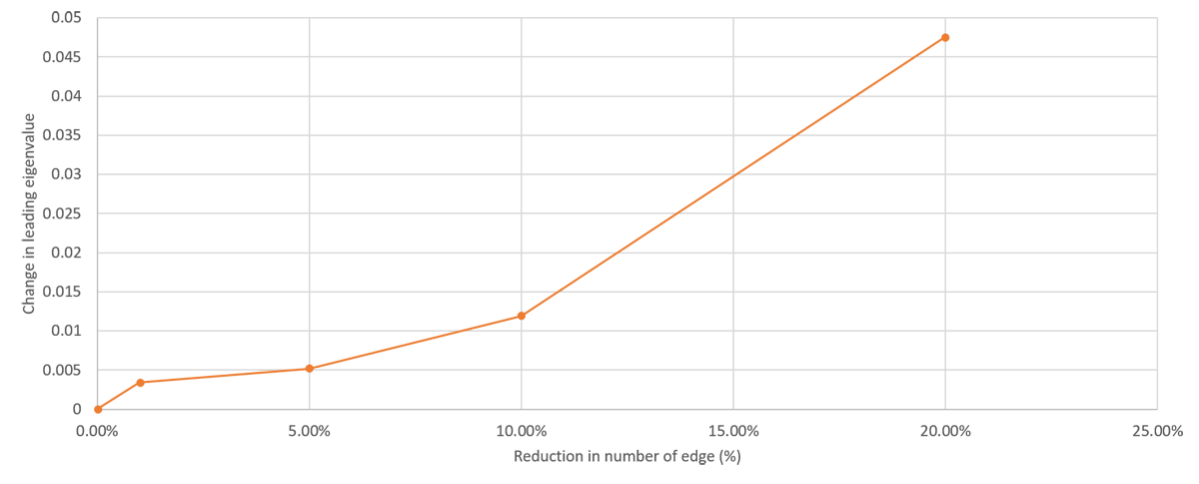
Decrease in the second eigenvalue with reduction in the number of edges.

**Figure 9:**
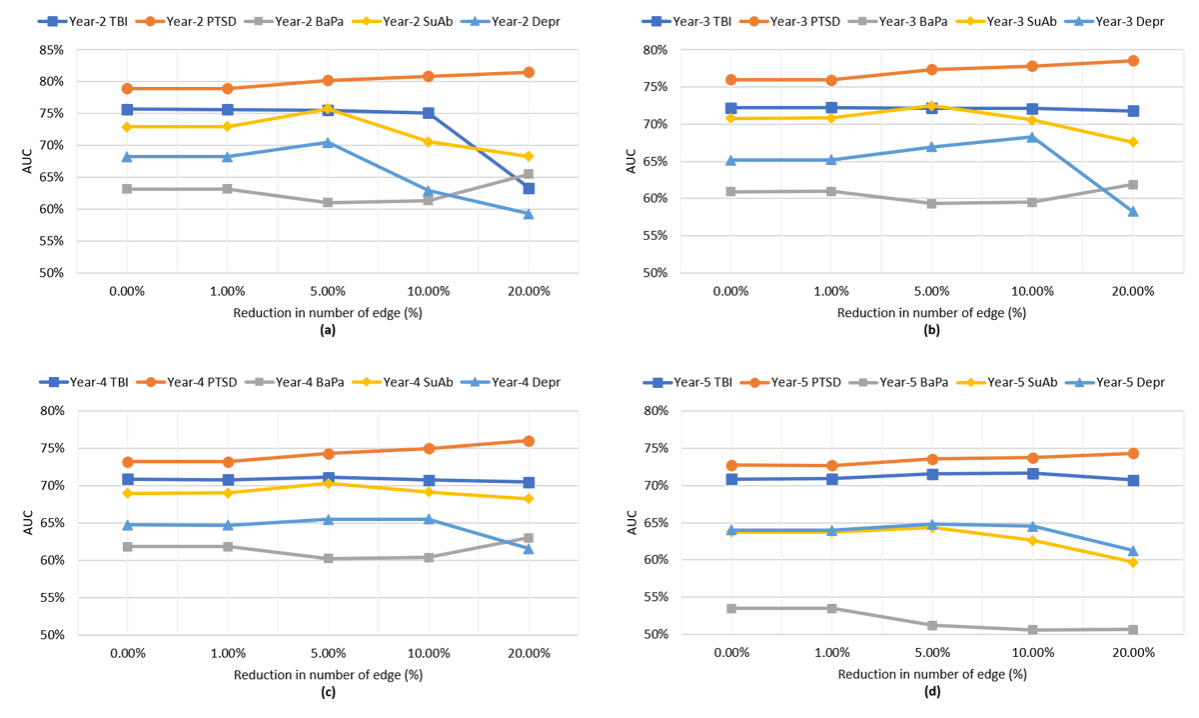
The relationship between the changes in the tuning parameters (λ) and the area under the curve in the second eigenvalue (λ) over (a) year-2, (b) year-3, (c) year-4, and (d) year-5 of the study. BaPa: back pain; TBI: traumatic brain injury; PTSD: posttraumatic stress disorder; SuAb: substance abuse; MCC: multiple chronic conditions; EAGL: eigenvalue analysis of the graph Laplacian.

Figure 8 presents the relationship between the various choices of compression ratio and the changes in the second eigenvalue of the graph Laplacian. As shown in the figure, for compression ratio values of >10%, the rate of change in the second eigenvalue increases. Moreover, Figure 9 provides the predictive accuracy of the summarized graph for the 5 chronic conditions in the study at different years (year 2 to year 5), which shows a reduction in the AUC for larger choices of summarization ratios (>10%). Therefore, a sharp increase in the changes in the second EAGL can be used as a stopping criterion for EAGL. (The analysis of the first eigenvalue is provided in Multimedia Appendix 1.)

### Summarizing a Graphical Model of Multiple Chronic Conditions Terms With No Supporting Data

**Figure 10:**
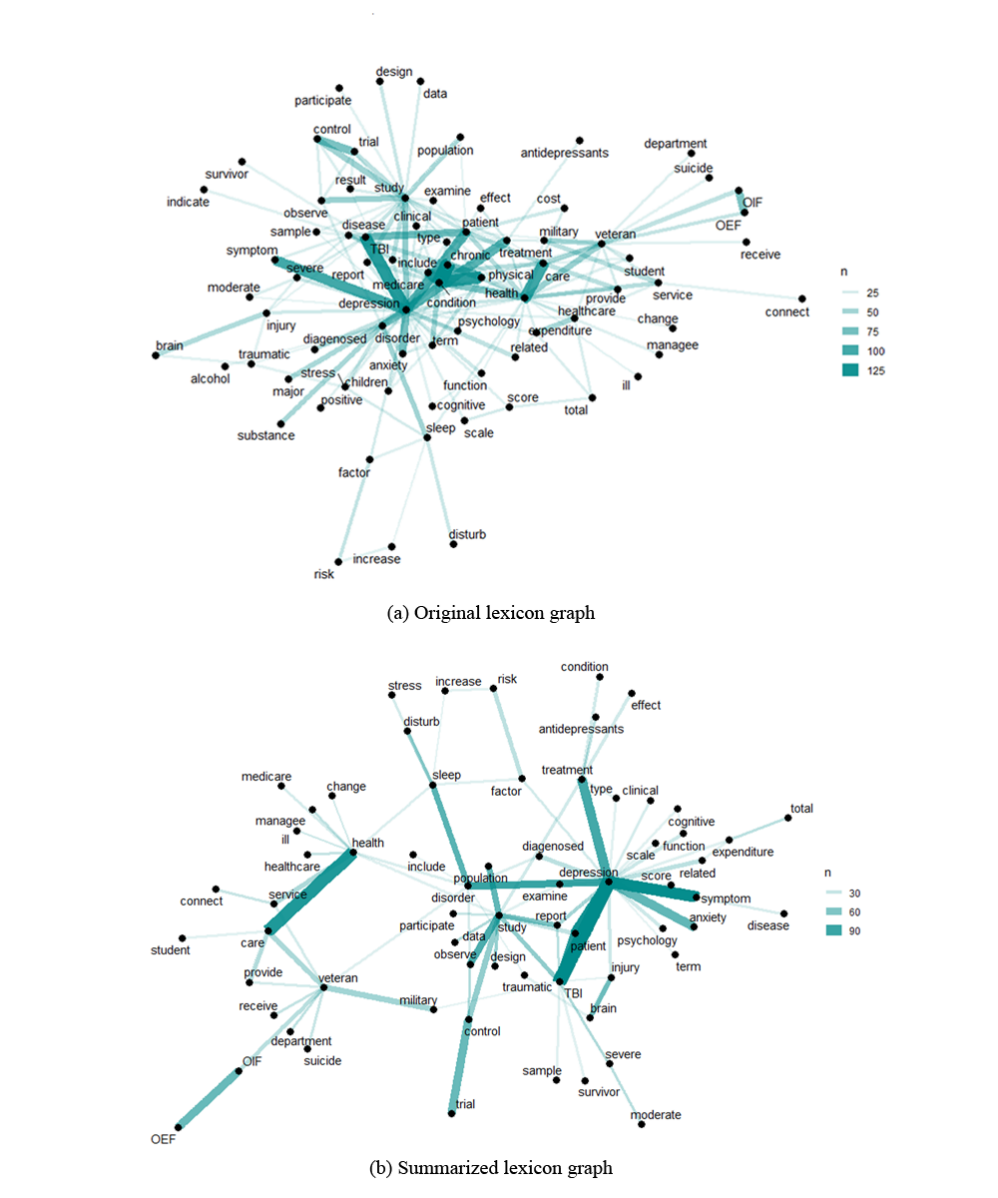
(a) Lexicon graph of the top 200 most frequent word pairs attained from text mining of 20 medical journal papers; (b) lexicon graph after summarization algorithm (70% summarization) was performed in the graph. OEF: operation enduring freedom; OIF: operation Iraqi freedom.

A lexicon graph contains a list of stems and affixes, together with basic information about them in the form of a graphical model. This is generally used to represent interconnected word pairs and their frequencies in natural language processing. Here, we are interested in exploring the opportunity to summarize a graphical model of MCC-related terms (Lexicon graph) with no supporting data using the EAGL algorithm. The graphical model was developed based on a lexicon graph from a collection of medical journals. The journals were extracted using the following keywords: Veterans, Traumatic Brain Injury, Back Pain, Post-Traumatic Stress Disorder, Depression, Substance Abuse, Chronic Diseases, Comorbidity, Multimorbidity, chronic conditions, chronic illness, and chronic pain. A total of 20 peer-reviewed journal papers were collected based on Google Scholar ranking (without expert opinion). Multimedia Appendix 3 lists the journal papers used for the creation of the lexicon graph. From the collected papers, the term and their frequencies are extracted and turned into a data set [41,62-81]. The 200 most frequent word pairs are then selected to build the lexicon graph, where the strength of the edges (connections) represents the co-occurrence of the word pairs in the same sentence (original lexicon graph in Figure 10).

Summarized lexicon graph in Figure 10 illustrates the summarized graphical model using the EAGL algorithm at a 70% summarization rate (edge removal) without utilizing any supporting data set. The summarized graph presents a cluster of strong relationships among chronic conditions such as <Depr, anxiety, TBI, symptoms, and treatment>. It also shows meaningful connections among <study, design, observe, population, control, and trial> and <healthcare, ill manage, service, and medicare>. There are also other interesting groups of highly connected terms such as <veteran care, military, suicide, and Operation Iraqi Freedom (OIF)> or <sleep, stress, and increased risk>. Multimedia Appendix 3 shows an enlarged version of the lexicon graph and its compressed form using the EAGL algorithm. It is worth noting that the algorithm here does not estimate/update the weight of (remaining) edges at each iteration (removal of edges); therefore, it is very efficient in summarizing large lexical graphs.

### Computational Complexity

In this section, we derive the time complexity of algorithms shown in Figures 4 and 5, which is presented earlier. Let *n* denote the number of node/variables/vertices (chronic conditions), *e* denote the number of edges (relationship between pair of chronic conditions), *m* denote the number of observations/cases (patient observations), and *r* denote the number of possible values/instances for each variable (in our study *r*=2, which represents having/not having a condition). Figure 4 consists of 5 components with the following (known) computational complexities: (1) MWST for node ordering: *0* (*n*^2^); (2) topological sorting: *0* (*n* + *e*); (3) graph Laplacian: *0* (*n*); (4) eigenvalue calculation: *0* (*n*^2^); and (5) K2 structure learning with regularization: *0* (*mn*^4^*r*). Integrating the complexities of the 5 components with some algebraic simplification, the overall complexity of Figure 4 can be derived as *0* (*mn*^4^*r*).

Figure 5 also consists of 3 components with the following (known) computational complexities: (1) depth-first tree extraction: *0* (*n* + *e*); (2) graph Laplacian: *0* (*n*); and (3) eigenvalue calculation: *0* (*n*^2^). Let *p* denote the number of edges to be removed (the desired amount of edge removal). After some algebraic operations (to account for the loops), the overall complexity of Figure 5 can be derived as *0* (*en*^2^*p*)

## Discussion

### Principal Findings

Graphical models are increasingly being used for descriptive, predictive, and prescriptive analytics in various applications, including social media, computer networks, genetics, and disease prognosis [7,8,82-84]. The effectiveness of a graphical model depends on the quality of the information propagating through nodes, which is affected by the topology of the network. Graph topology also affects other properties of a graphical model, including complexity, robustness, and scalability [85]. A fully connected network can be considered the most robust in terms of information dissemination but may cause overfitting, slow training, and memory allocation issues. Graph summarization can be performed to identify the important structures, major patterns, and dissemination of information in complex graphical models of MCC interaction.

In this study, we have addressed the problem of summarizing complex graphical models and identifying their important patterns by modifying the edges of the graph. These types of graphical frameworks are useful for analyzing plausible interactions between disease states [4]. The eigenvalue of the graph Laplacian reveals the characteristics of a graph. For a large graph, the second eigenvalue of the graph Laplacian determines the amount of information that is being distributed by the graph. Thus, by analyzing the second eigenvalue of the graph Laplacian, we attain a measure (EAGL) of sparse cutoff. The proposed EAGL algorithm can be used as an active learning unsupervised method to directly learn a sparse probabilistic graphical model from an available data set or summarize an existing graphical model with or without a supporting data set.

The first approach (using direct learning) results in a refined model where network analysis can be performed by an end user with specific needs and expertise. Our direct learning model (Figure 4) demonstrates very good performance when data are available, and the algorithm is able to learn de novo. This results in a graph (Figure 1) with predictive abilities that can be interpreted by clinicians and medical researchers with an understanding of the medical conditions of interest.

The second approach (Figure 5) summarizes an existing graphical model with or without a supporting data set. The EAGL algorithm, which is based on a simplification-based rule edge removal strategy, can also be used to reveal important patterns within a given graphical model by removing the edges with a marginal contribution to the leading eigenvalue of the graph Laplacian.

Our findings revealed that the proposed summarization algorithm can indeed improve the predictive accuracy of the summarized graphical model while reducing its size and increasing the inference efficiency. We used 2 data sets of (1) 257,633 veteran patients who have been monitored for the emergence of 5 multiple conditions (TBI, PTSD, BaPa, Dep, and SuAb) over 5 years and (2) the coappearance of the 200 most frequent word pairs in the literature of MCC to validate the performance of the proposed EAGL approach.

Although the statistical details of the proposed model might be complex for some practitioners to understand, the resulting algorithm can be seen as a step toward creating more interpretable analytical models for understanding the evolution of MCC, by removing less informative edges in complex networks of MCC (resulting in a sparser network), without losing predictive accuracy. In fact, practitioners do not need to know the details of the proposed algorithm to utilize it. They can use a simple tuning parameter (λ) to control the level of resulting network sparsity (number of remaining edges), that is, setting a high value for the tuning parameter results in a very sparse network (with few edges), which is easy to understand (Figures 1 and 2). Such a (sparse) graphical representation provides a straightforward visualization of how the presence of one condition can affect the emergence of another condition without complex statistics. It also helps interpret the probabilistic results from statistical analysis.

Finally, the proposed EAGL approach can help medical practitioners and health care analysts not only in terms of developing a predictive tool to analyze the probability of a new chronic condition development, given the existing conditions (Figures 6-9), but also by using a tuning parameter (λ) to identify major interaction patterns among MCC. The model can also be used as a visualizing tool to inspect the interaction among MCC (Figures 1 and 2).

### Limitations

Although the proposed EAGL algorithm successfully extracts important connections and controls the level of sparsity, it has a few limitations and potential problems. Algorithm presented in Figure 4 needs to be built on top of a structure learning model. In this study, we utilized the MWST + K2 method [9]. This is a heuristic-based structure learning model, where the initial node order has to be known or learned using the MWST method. Algorithm presented in Figure 5 requires an appropriate tree extraction method to ensure that there will be no island node (or set of nodes), which can limit the level of summarization. In addition, for a high summarization ratio, the summarization algorithm can decrease the prediction accuracy. Finally, both algorithms (Figures 4 and 5) primarily target acyclic graphs, but their usefulness to depict complex webs of causation in chronic conditions, which can involve loops (particularly of reinforcing types), is limited.

### Conclusions

In this work, we propose a graph summarization approach that utilizes the second eigenvalue of the graph Laplacian to identify and prune less informative edges of the complex graphical models of MCC interaction. We developed 3 algorithms based on the proposed approach to deal with different scenarios with respect to the availability of data and/or a graphical model. The first algorithm learns a sparse graphical model of MCC interactions directly from the data by regularizing an existing score-based structure learning algorithm to achieve a desired level of sparsity. The second algorithm summarizes an existing graph of MCC interactions by removing less informative connections with respect to a supporting data set. The third algorithm simplifies a given MCC graph by removing the less important edges without a supporting data set. We validated the performance of the first 2 algorithms based on a large data set of veteran patients who have been monitored for over 5 years and 5 multiple chronic medical conditions, including PTSD, TBI, Depr, BaPa, and SuAb. We also validated the third algorithm based on a data set of coappearances of the 200 most frequent word pairs in the literature of MCC. The results showed that the proposed EAGLE algorithm effectively extracts important connections and dependency patterns from the complex graphical model of the interactions of MCC. It can also control the level of sparsity in the resulting graph based on the practitioners’ needs using a simple tuning parameter. Finally, it improves the predictive accuracy of the resulting summarized graphical model.

## Appendix

Multimedia Appendix 1 Results of the eigenvalue analysis of the graph Laplacian algorithm based on the first eigenvalue.

Multimedia Appendix 2 A sample example of the eigenvalue analysis of the graph Laplacian algorithm for a small graph.

Multimedia Appendix 3 Multiple chronic conditions term lexicon.

## Abbreviations

AIC: Akaike information criterion
AUC: area under the curve
BaPa: back pain
BN: Bayesian network
DAG: directed acyclic graph
Depr: depression
DFS: depth-first search
EAGL: eigenvalue analysis of the graph Laplacian
MCC: multiple chronic conditions
MWST: maximum weight spanning tree
PTSD: posttraumatic stress disorder
ROC: receiver operating characteristic
SuAb: substance abuse
TBI: traumatic brain injury
VA: Veterans Health Administration
