# Supplementary material for: Summarizing Complex Graphical Models of Multiple Chronic Conditions Using the Second Eigenvalue of Graph Laplacian: Algorithm Development and Validation
Source: JMIR Med Inform. 2020 Jun 17;8(6):e16372. doi: 10.2196/16372 (PMC7330739; doi:10.2196/16372)

### Multimedia Appendix 1: Results of EAGL Algorithm Based on First Eigen Value

#### 1.1 Learning sparse probabilistic graphical models directly from (MCC) data:

Table 1: The area under the curve (AUC) performance of the sparse probabilistic graphical model learned by the EAGL algorithm (based on the 1^st^ eigenvalue) directly from the data with different choices of tuning parameters ($\lambda=0, {10}^{-2},{10}^{-1},\ldots, {10}^{5}$) for predicting future comorbidities (year-2 to year-5), given comorbidity information of the past year (year-1), along with AUC performance of a comparing algorithm, namely Akaike information criterion (AIC) (the bottom row), as well as associated summarization ratios (the right-hand side column).


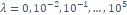


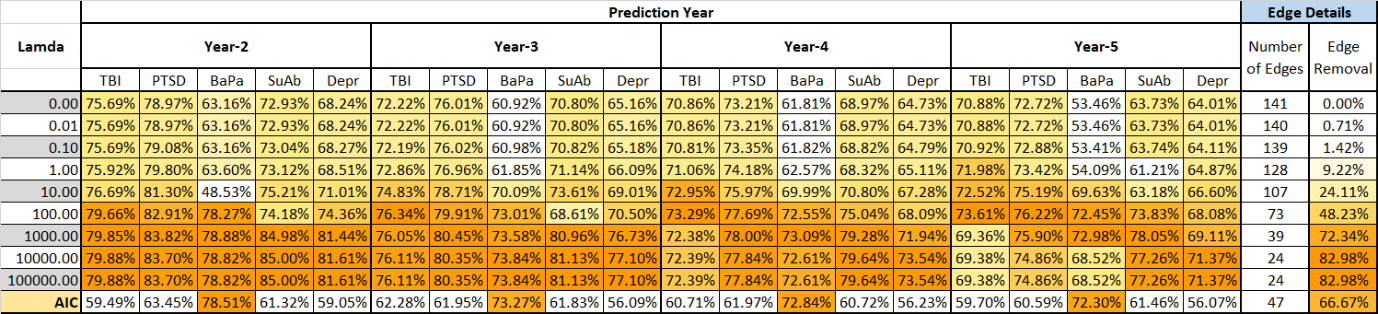


#### 1.2 Summarizing an existing (MCC) probabilistic graphical model with supporting data:

Table 2: The Area Under the Curve (AUC) performance of the original and summarized probabilistic graphical models using EAGL algorithm (based on the 1^st^ algorithm) at different summarization ratios (1%, 5%, 10%, 20%) for predicting future comorbidities (year-2 to year-5), given comorbidity information of the past year (year-1).


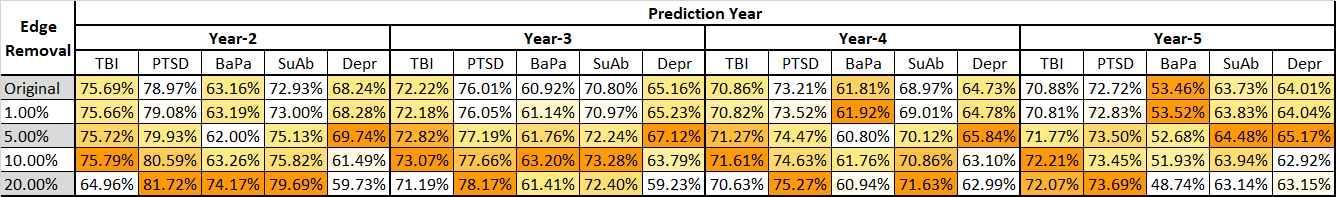

Supplement: Multimedia Appendix 1 [file medinform_v8i6e16372_app1.docx]
