# Supplementary material for: Summarizing Complex Graphical Models of Multiple Chronic Conditions Using the Second Eigenvalue of Graph Laplacian: Algorithm Development and Validation
Source: JMIR Med Inform. 2020 Jun 17;8(6):e16372. doi: 10.2196/16372 (PMC7330739; doi:10.2196/16372)
Supplement: Multimedia Appendix 2 [file medinform_v8i6e16372_app2.docx]

### Multimedia Appendix 2: A Sample Example of EAGL Algorithm for a Small Graph

Figure 2.1 demonstrates a visual representation of the EAGL algorithm for summarizing the disease-disease interaction graph (TBI: Traumatic Brain Injury, PTSD: Post Traumatic Stress Disorder, SuAb: Substance Abuse, BaPa: Back Pain, Depr: Depression). The graph was attained from data using the Bayesian structure learning method. Given a limit of k = 3 edges to remove, at each iteration it checks the desired criteria and removes an edge that minimizes the leading eigenvalue. Once it reaches the allocated threshold, the algorithm returns the final graph. For summarizing this graph, we have adopted One Edge at a time approach.


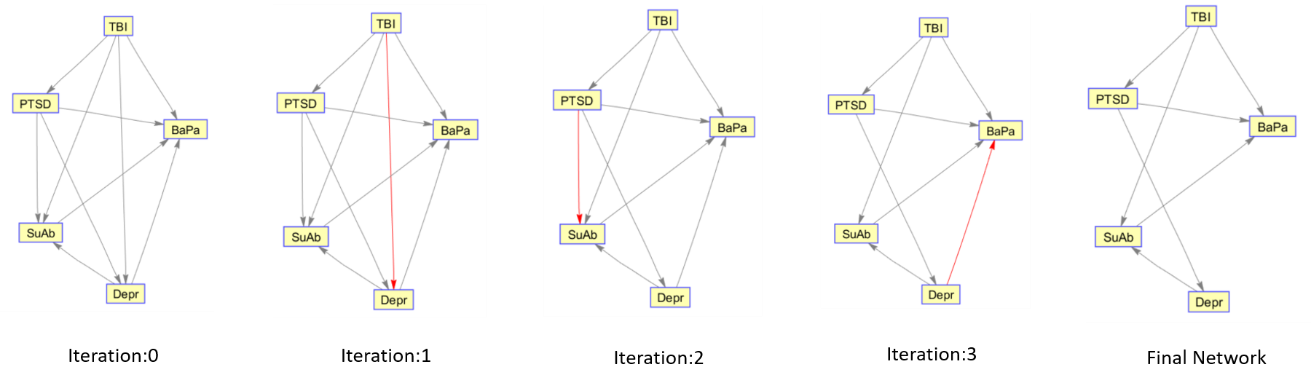


Figure 2.1: A sample example of EAGL algorithm applied in a small disease interaction graph
