# Supplementary material for: Summarizing Complex Graphical Models of Multiple Chronic Conditions Using the Second Eigenvalue of Graph Laplacian: Algorithm Development and Validation
Source: JMIR Med Inform. 2020 Jun 17;8(6):e16372. doi: 10.2196/16372 (PMC7330739; doi:10.2196/16372)
Supplement: Multimedia Appendix 3 [file medinform_v8i6e16372_app3.docx]

### Multimedia Appendix 3: Multiple Chronic Condition Term Lexicon

Table 3.1 shows the list of paper used for the creation of a lexicon graph. The journals are extracted using the keywords: *Veterans, Traumatic Brain Injury, Back Pain, Post-Traumatic Stress Disorder, Depression, Substance Abuse, Chronic Diseases, Comorbidity, Multimorbidity, chronic conditions, chronic illness, and chronic pain* in Google Scholar.

Table 3.1: List of Papers used for creating the graphical model for NLP [41,62–81].

| **Author** | **Title** | **Journal** |
| --- | --- | --- |
| Bay, E., et al., | Chronic stress, sense of belonging, and depression among survivors of traumatic brain injury. | Journal of Nursing Scholarship, 2002. 34(3): p. 221-226. |
| Bhattacharya, R., et al., | Depression treatment decreases healthcare expenditures among working age patients with comorbid conditions and type 2 diabetes mellitus along with newly diagnosed depression. | BMC psychiatry, 2016. 16(1): p. 247. |
| Bramoweth, A.D., et al., | Identifying the demographic and mental health factors that influence insomnia treatment recommendations within a veteran population. | Behavioral sleep medicine, 2019. 17(2): p. 181-190. |
| Concato, J., et al. | Randomized, controlled trials, observational studies, and the hierarchy of research designs. | New England Journal of Medicine, 2000. 342(25): p. 1887-1892. |
| Corson, K., et al., | Prevalence and correlates of suicidal ideation among Operation Enduring Freedom and Operation Iraqi Freedom veterans. | Journal of Affective Disorders, 2013. 149(1-3): p. 291-298. |
| Diederichs, C., et al. | The measurement of multiple chronic diseases—a systematic review on existing multimorbidity indices. | Journals of Gerontology Series A: Biomedical Sciences and Medical Sciences, 2010. 66(3): p. 301-311. |
| Feinstein, A.R., | The pre-therapeutic classification of co-morbidity in chronic disease. | Journal of chronic diseases, 1970. 23(7): p. 455-468. |
| Fenton, B.T., et al., | Relationships between temporomandibular disorders, MSD conditions, and mental health comorbidities: findings from the veteran’s musculoskeletal disorders cohort. | Pain Medicine, 2018. 19(suppl_1): p. S61-S68. |
| Glenn, M.B., et al., | Depression amongst outpatients with traumatic brain injury. | Brain injury, 2001. 15(9): p. 811-818. |
| Gunn, J.M., et al., | The association between chronic illness, multimorbidity and depressive symptoms in an Australian primary care cohort. | Social psychiatry and psychiatric epidemiology, 2012. 47(2): p. 175-184. |
| Haibach, J.P., et al., | Military and veteran health behavior research and practice: challenges and opportunities. | Journal of behavioral medicine, 2017. 40(1): p. 175-193. |
| Hunter, G., et al., | Health care utilization patterns among high-cost VA patients with mental health conditions. | Psychiatric Services, 2015. 66(9): p. 952-958. |
| Magnavita, N. and S. Garbarino, | Sleep, health and wellness at work: A scoping review. | International journal of environmental research and public health, 2017. 14(11): p. 1347. |
| Mastrocola, E.L., et al. | Access to healthcare for long-term conditions in women involved in street-based prostitution: a qualitative study. | BMC family practice, 2015. 16(1): p. 118. |
| McGlinchey, R.E., et al., | A methodology for assessing deployment trauma and its consequences in OEF/OIF/OND veterans: The TRACTS longitudinal prospective cohort study. | International journal of methods in psychiatric research, 2017. 26(3): p. e1556. |
| Peterson, J., et al. | An interprofessional education project to address veterans’ healthcare needs. | International Journal of Higher Education, 2016. 6(1): p. 1. |
| Rosenthal, M., et al. | Depression following traumatic brain injury. | Archives of physical medicine and rehabilitation, 1998. 79(1): p. 90-103. |
| Swartz, J.A., | Chronic medical conditions among jail detainees in residential psychiatric treatment: a latent class analysis. | Journal of Urban Health, 2011. 88(4): p. 700-717. |
| Thabrew, H., et al., | Psychological therapies for anxiety and depression in children and adolescents with long‐term physical conditions. | Cochrane Database of Systematic Reviews, 2018(12). |
| Zis, P., et al., | Depression and chronic pain in the elderly: links and management challenges. | Clinical interventions in aging, 2017. 12: p. 709. |


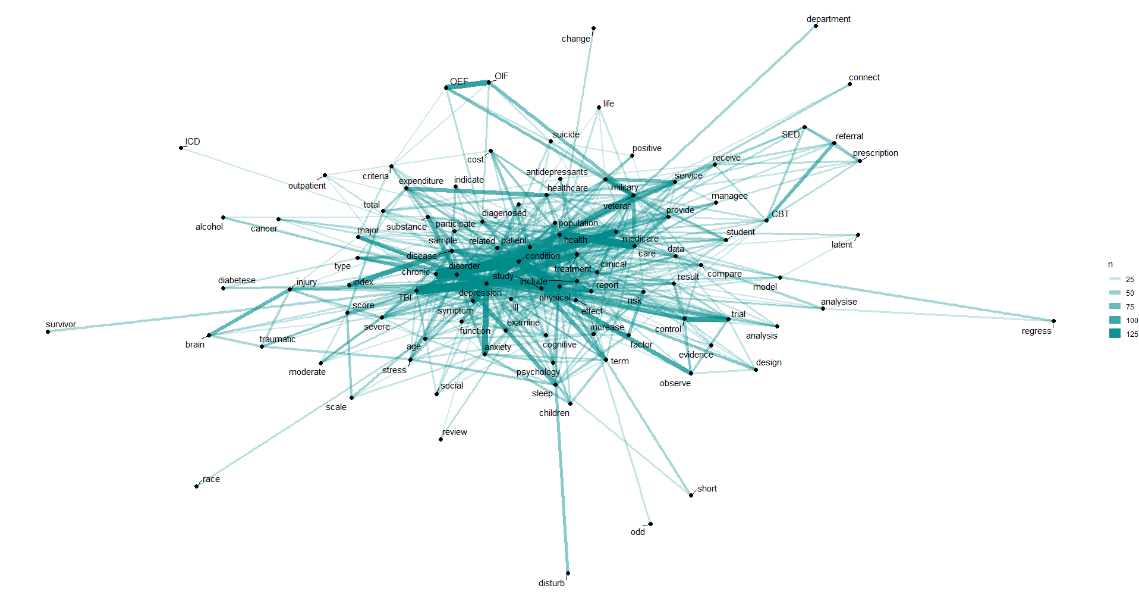


(a)


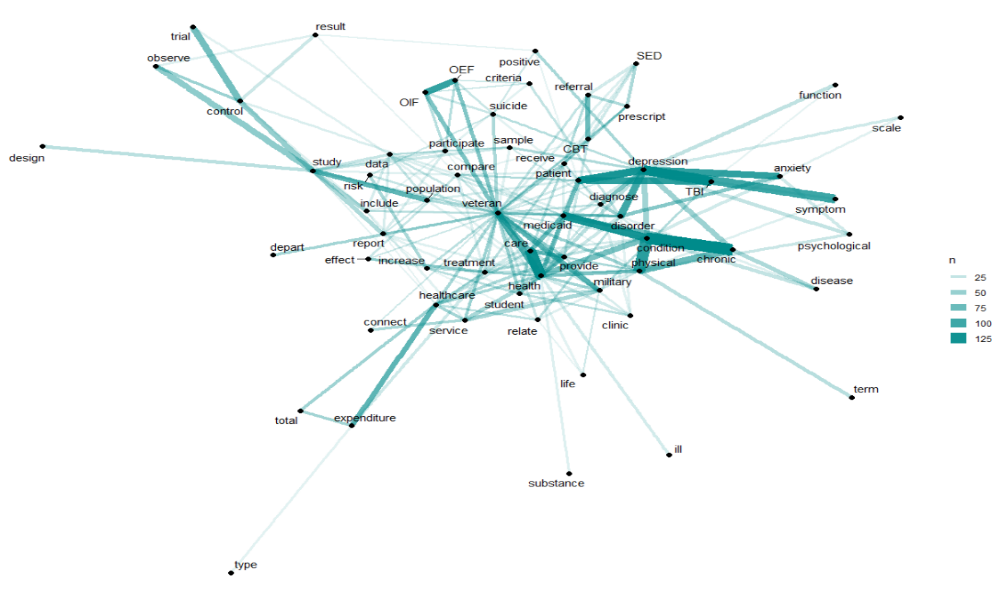


(b)

Figure 3.1.: (a) Lexicon Graph of the top 100 frequent words attained from text mining of 20 medical journal papers; (b) Lexicon Graph after summarization algorithm (70% summarization rate) was performed in the graph.
